# Whole genome sequencing of multidrug-resistant *Salmonella enterica* serovar Typhimurium isolated from humans and poultry in Burkina Faso

**DOI:** 10.1186/s41182-018-0086-9

**Published:** 2018-02-12

**Authors:** Assèta Kagambèga, Taru Lienemann, Jonathan G. Frye, Nicolas Barro, Kaisa Haukka

**Affiliations:** 1 Bacteriology Unit, Department of Infectious Disease Surveillance and Control, National Institute for Health and Welfare (THL), Helsinki, Finland; 2 Laboratoire de Biologie Moléculaire, d’épidémiologie et de surveillance des bactéries et virus transmissibles par les aliments (LaBESTA)/Centre de Recherche en Sciences Biologiques, Alimentaires et Nutritionnelles (CRSBAN)/Ecole Doctorale Sciences et Technologies (EDST)/Université Ouaga I Professeur Joseph KI-ZERBO, 03 BP 7021, Ouagadougou, 03 Burkina Faso; 3 Institut Des Sciences (IDS), 01 BP 1757, Ouagadougou, 01 Burkina Faso; 4 Department of Food and Environmental Sciences, Division of Microbiology and Biotechnology, University of Helsinki, P.O.Box 56, FI-00014 Helsinki, Finland; 5 Bacterial Epidemiology and Antimicrobial Resistance Research Unit, US National Poultry Research Center, US Department of Agriculture, Agricultural Research Service, Athens, GA USA

**Keywords:** MDR *Salmonella Typhimurium*, WGS, Human feces, Poultry feces, Burkina Faso

## Abstract

**Background:**

Multidrug-resistant *Salmonella* is an important cause of morbidity and mortality in developing countries. The aim of this study was to characterize and compare multidrug-resistant *Salmonella enterica* serovar Typhimurium isolates from patients and poultry feces.

**Methods:**

*Salmonella* strains were isolated from poultry and patients using standard bacteriological methods described in previous studies. The strains were serotype according to Kaufmann-White scheme and tested for antibiotic susceptibility to 12 different antimicrobial agents using the disk diffusion method. The whole genome of the *S.* Typhimurium isolates was analyzed using Illumina technology and compared with 20 isolates of *S.* Typhimurium for which the ST has been deposited in a global MLST database.The ResFinder Web server was used to find the antibiotic resistance genes from whole genome sequencing (WGS) data. For comparative genomics, publicly available complete and draft genomes of different *S.* Typhimurium laboratory-adapted strains were downloaded from GenBank.

**Results:**

All the tested *Salmonella* serotype Typhimurium were multiresistant to five commonly used antibiotics (ampicillin, chloramphenicol, streptomycin, sulfonamide, and trimethoprim). The multilocus sequence type ST313 was detected from all the strains. Our sequences were very similar to *S.* Typhimurium ST313 strain D23580 isolated from a patient with invasive non-typhoid *Salmonella* (NTS) infection in Malawi, also located in sub-Saharan Africa. The use of ResFinder web server on the whole genome of the strains showed a resistance to aminoglycoside associated with carriage of the following resistances genes: *strA*, *strB*, and *aadA1*; resistance to β-lactams associated with carriage of a *bla*_*TEM-1B*_ genes; resistance to phenicol associated with carriage of *catA1* gene; resistance to sulfonamide associated with carriage of *sul1* and *sul2* genes; resistance to tetracycline associated with carriage of *tet B* gene; and resistance to trimethoprim associated to *dfrA1* gene for all the isolates*.*

**Conclusion:**

The poultry and human isolates were genetically similar showing a potential food safety risk for consumers. Our finding of multidrug-resistant *S.* Typhimurium ST313 in poultry feces calls for further studies to clarify the potential reservoirs of this emerging pathogen.

## Background

*Salmonella enterica* is one of the leading causes of zoonotic foodborne disease worldwide. The global burden of diarrheal disease caused by S*almonella* gastroenteric infections is substantial, with estimated 93.8 million human cases per year and 155,000 deaths [1]. These diseases are particularly frequent among children in developing countries such as Burkina Faso, where they often go unreported because of the lack of the foodborne pathogens surveillance system. In addition to causing diarrheal disease, *Salmonella* cause invasive bloodstream infections, particularly in sub-Saharan Africa [2]. A distinct genotype of invasive multiple-antibiotic-resistant *S.* Typhimurium, ST313, has been identified as an emerging pathogen causing life-threatening infections especially to adults with HIV infection and children suffering from malnutrition and malaria [2, 3]. Also in Burkina Faso, malaria combined with invasive bacterial infection caused by antibiotic-resistant *Salmonella* has been found to be related to high case fatality rate among children [4]. The source of the invasive infections and the possible role of animals spreading the pathogen are not well known.

We have previously studied *Salmonella* strains isolated from feces of diarrheal children under 5 years old and from feces of animals, including poultry [5, 6]. Seventeen (17) multidrug-resistant *S.* Typhimurium isolates from human and chicken feces were compared by pulsed-field gel electrophoresis and found to closely resemble each other genetically. Some of them were resistant to multiple commonly prescribed antimicrobials (ampicillin, chloramphenicol, streptomycin, sulfonamides, and trimethoprim). The aims of this study were to investigate the genomes of the multidrug-resistant (MDR) *S.* Typhimurium strains isolated from the feces of humans and poultry in Burkina Faso using whole genome sequencing (WGS) and to compare the sequences to each other as well as to other previously published genomes of MDR *S.* Typhimurium.

## Methods

### Bacterial strains

The strains used in this study have been described in our previous studies [5, 6]. The strain 96069 was isolated from a 4-year-old boy and 96071 from a 2-year-old girl, both with diarrhea in Ouagadougou, the capital city of Burkina Faso. The strains 98233 and 98983 were isolated from the feces of slaughtered poultry. The human strains were of phage type DT2 and the poultry strains of DT56 [6].

### DNA extraction and whole genome sequencing

Genomic DNA was isolated using the MaqAttract® kit (Qiagen, UK). DNA quantity and quality were analyzed using gel electrophoresis and the Qubit® device (Invitrogen, USA). Library preparation was done using Nextera XT DNA Library Preparation Kit (Illumina, San Diego, CA, USA). Sequencing was performed with a MiSeq benchtop sequencer (Illumina, San Diego, CA, USA).

### De novo genome assembly and MLST

Whole genome sequences (WGS) of *S.* Typhimurium isolates from the feces of two patients with diarrhea and two slaughtered poultry from Ouagadougou, capital city of Burkina Faso, were analyzed by multilocus sequence typing (MLST). De novo assembly was performed using Velvet assembler included in the Ridom SeqSphere+ software. The sequencing reads were trimmed before assembly using default settings of Ridom SeqSphere+ (trim at both ends of the reads until the average base quality was > 30 in a window of 20 bases). An automatic k-mer mode was used to define the best k-mer value to be used in the assembly. The UPGMA dendrogram was constructed using the allele call results of the core genome MLST (cgMLST) targets. For comparative genomics, the obtained sequences of strains from human and poultry feces were compared to each other and to complete or draft genome sequences of 20 *S.* Typhimurium strains downloaded from Genbank (http://www.ncbi.nlm.nih.gov/genbank).

### Antimicrobial resistance testing

The *S.* Typhimurium strains were retested for antibiotic susceptibility to 12 different antimicrobial agents using the disk diffusion method on Mueller-Hinton agar (Oxoid) at 37 °C for 24 h. The antibiotic disks (Oxoid) used were ampicillin (10 μg), chloramphenicol (30 μg), streptomycin (10 μg), sulfonamide (300 μg), trimethoprim (5 μg), ciprofloxacin (5 μg), tetracycline (30 μg), gentamicin (10 μg), nalidixic acid (30 μg), cefotaxime (5 μg), mecillinam (10 μg), and imipenem (10 μg).

### Identification of resistance genes

The ResFinder web server (http://www.genomicepidemiology.org) was used to identify antimicrobial resistance genes in the WGS data, using a threshold of 98.00% identity (ID). The ResFinder results were compared with phenotypic antimicrobial susceptibility testing.

## Results

**Fig. 1:**
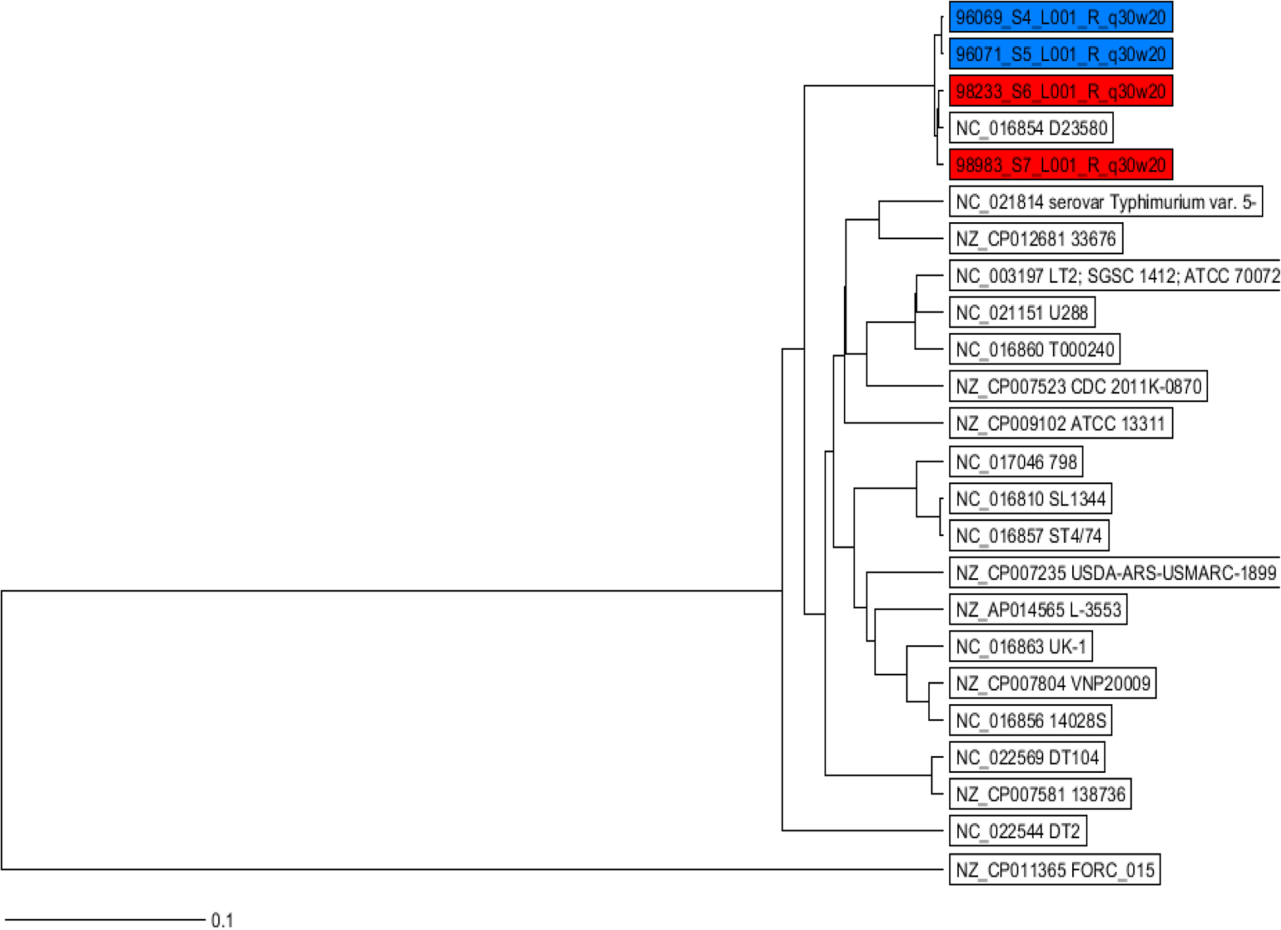
UPGMA tree showing comparison of the MLST sequences of four Burkinabe and 20 reference strains. The tree is based on 3214 columns, no missing values, and % column difference. The human isolates from Burkina Faso are highlighted with blue and chicken isolates with red

The MLST analysis of the WGS data revealed that all the isolates were of the same sequence type, ST313. Our sequences were very similar to *S.* Typhimurium ST313 strain D23580 downloaded from Genbank, which was isolated from a patient with invasive non-typhoid *Salmonella* (NTS) infection in Malawi, also located in sub-Saharan Africa (Fig.1). The *S.* Typhimurium isolates described in this study were resistant to five antimicrobials (ampicillin, chloramphenicol, streptomycin, sulfonamide, and trimethoprim) using phenotypical method. Resistance to tetracycline was not detected by the disk diffusion method. The ResFinder web server was used to find the antimicrobial resistance genes in the isolates’ sequences. Genes conferring resistance to six classes of antimicrobials were detected in the genomes of the isolates. Resistance to aminoglycoside was associated with carriage of the following resistances genes: *strA*, *strB*, and *aadA1*; resistance to β-lactams was associated with carriage of a *bla*_*TEM-1B*_ gene; resistance to chloramphenicol was associated with carriage of *catA1* gene; resistance to sulfonamide was associated with carriage of *sul1* and *sul2* genes; and resistance to trimethoprim was associated to *dfrA1* gene for all the isolates. The *tetB* gene, which usually confers resistance to tetracycline, was also detected; however, phenotypic resistance to tetracycline was not detected (Table 1).

**Table 1:** Resistance genes in the four Burkinabe isolates detected with ResFinder

Resistance gene present	Confer resistance to the class of antimicrobials	Detected resistance by disk method
*aadA1*	Aminoglycosides	Streptomycin
*strB*		
*strA*		
*blaTEM-1B*	Beta-lactam	Ampicillin
*catA1*	Phenicol	Chloramphenicol
*sul1*	Sulfonamide	Sulfonamide
*sul2*		
*tetB*	Tetracycline	Not detected
*dfrA1*	Trimethoprim	Trimethoprim

## Discussion

Multidrug-resistant *S.* Typhimurium is a common cause of invasive NTS disease in sub-Saharan Africa [7, 8]. In this study, the sequenced strains of *S.* Typhimurium ST313 were from the bacteria isolated from the feces of patients with diarrhea and from slaughtered poultry. This is the first study reporting finding of ST313 from Burkina Faso, but it has been reported from several other sub-Saharan countries such as Malawi, Kenya, Nigeria, and Democratic Republic of Congo [9–12]. Strain D23580 from Malawi has served as a representative of the lineage presently causing an epidemic in Africa [3, 13]. In addition, this strain was the only ST313 among the 20 *S.* Typhimurium strains downloaded from the *S. enterica* MLST database and was isolated from a case of invasive NTS disease in Malawi [9]. The dominant sequence type from the 20 strains in MLST database was ST19 (60%), to which most of the sequenced strains isolated from Europe and America belong.

Recent studies have indicated that transmission of ST313 takes place mainly from person to person, but the possible transmission from animals to humans has not been excluded [14]. Lately, it has been shown that ST313 can cause severe invasive infection in chickens [15], in mice, and in monkeys [13]. Our sequencing results show that ST313 isolates can be obtained from chicken feces slaughtered at the common marketplace in Ouagadougou as well as from the feces of local children with diarrhea. It will be necessary to extend the study to other animals to know the possible host range of invasive *Salmonella* strains in Africa. These findings support the necessity of the one health promotion in all countries from Africa particularly against zoonotic pathogens like *Salmonella*.

The following resistance genes were found from the WGS data of the four *S.* Typhimurium strains in this study: *strA*, *strB*, and *aadA1*; *bla*_*TEM-1B*_; *catA1*; *sul1* and *sul2*; *tet B*; *dfrA1* with resistance pattern ACSSuT. In many regions of Burkina Faso as elsewhere in Africa, traditional poultry breeding is still prevalent, allowing the poultry to roam freely in the household with people [6, 16]. Thus, the shared living space and consumer’s preference for poultry products combined with poor understanding of hygiene seem to be the main cause of human infections. In larger-scale poultry production also in Africa, the misguided use of antibiotics for the treatment of animals and as growth promoters is an increasing problem possibly causing antibiotic resistance in pathogenic bacteria [17]. Uncontrolled use of antibiotics can cause selection for bacterial resistance posing a risk for public health by spreading of the resistance from farm animals to the human population [7]. The same resistance pattern ACSSuT has been found in MDR *S.* Typhimurium strain in many geographical areas [18, 19]. This resistance pattern is a typical one found in the ST313 with a sub-Saharan distinct genotype causing epidemic invasive disease. Recently, fluoroquinolone was highly active against enteropathogens, for example, in Africa; ciprofloxacin was the drug of choice for patients with enteric infection, which is very common in sub-Saharan Africa [20]. The misuse of ciprofloxacin creates resistance among enteric pathogens especially *Salmonella enterica*, which is the primary cause of enteric infection in Africa and is generally showing multiple drug resistance. However, in sub-Saharan Africa, ciprofloxacin is still the most commonly prescribed fluoroquinolone along with levofloxacin, ofloxacin, and norfloxacin.

In this study, all the strains carried the TEM-type ESBL (extended-spectrum beta-lactamases) and are co-resistant to four other antibiotics. This is in accordance with an earlier notion that multidrug resistance of non-typhoidal *Salmonella* involves the acquisition and accumulation of plasmid-mediated resistance determinants and/or spreading of mobile elements, such as transposons [21]. The ability of these mobile elements to transfer within or between bacterial species is associated with the rapid spread of antimicrobial resistance among the *Enterobacteriaceae*, including *Salmonella* [22, 23]. This finding is worrying because the ESBLs are lactamases capable of conferring bacterial resistance to the penicillins; to first-, second-, and third-generation cephalosporins; and to aztreonam by hydrolysis of these antibiotics, they may be inhibited by lactamase inhibitors such as clavulanic acid [18].

The present study revealed the need for a good surveillance system of the use of antibiotics in human and veterinary medicine. Development of awareness programs to control the unregulated use of antibiotics and self-medication for the treatment of infections in sub-Saharan countries will also contribute to the reduction of resistance problems and prolong the effectiveness of the new generation antibiotics. The spread of multidrug resistant *S.* Typhimurium can pose a threat to the management of salmonellosis in animal husbandry and human medicine. It will be a great problem for human medicine to deal with untreatable *Salmonella* infections due to the lack of effective antimicrobials.

## Conclusion

In this study, we described the whole genome sequencing results of MDR *S.* Typhimurium strains isolated from the feces of humans and poultry in Burkina Faso. This study provides new data to better understand the geographical spread of *S.* Typhimurium ST313 genotypes and their potential host reservoirs in sub-Saharan Africa. Our future plan is to isolate more MDR ST313 strains from various sources in Burkina Faso, submit them to WGS, and analyze the sequences in detail in order to better understand the linkage between the environment, host animals, and the human infections caused by ST313. The epidemic caused by *S.* Typhimurium in sub-Saharan Africa shows that next-generation sequencing facilities should be available also to the scientists in the resource-limited African countries to detect epidemics in their early phase. The present study highlights the need of a good surveillance system of foodborne pathogens to prevent enteric diseases in sub-Saharan Africa.
